# Differential temporal dynamics in motor imagery shaped by agent type and action duration

**DOI:** 10.1007/s00426-026-02259-9

**Published:** 2026-02-14

**Authors:** Lorenzo Viviani, Alba Liso, Lisa Colotti, Sara Romano, Valentina Fogo, Fabrizio Riguzzi, Giovanni Buccino, Laila Craighero

**Affiliations:** 1 Department of Medical Sciences, University of Ferrara, via Fossato di Mortara 64/B, Ferrara, 44121 Italy; 2 Department of Neuroscience and Rehabilitation, University of Ferrara, via Fossato di Mortara 19, Ferrara, 44121 Italy; 3 Department of Mathematics and Computer Science, University of Ferrara, via Machiavelli 35, Ferrara, 44121 Italy; 4 Faculty of Medicine and Surgery, University “Vita-Salute” San Raffaele, IRCCS San Raffaele, Milan, Italy

**Keywords:** Motor Imagery, Robotic Actions, Temporal Judgment, Action Duration, Human-RobotInteraction, Representational momentum

## Abstract

This study explored the temporal dynamics of motor imagery (MI) following the observation of robotic versus human actions using a temporal judgment task. Participants observed goal-directed action videos by a human or a NAO robot, performed MI until an auditory stop signal, then completed a two-alternative forced choice task to identify the frame corresponding to their imagery stop point, choosing between the correct frame and either a preceding (‘Before’) or succeeding (‘After’) frame. Results revealed a systematic temporal bias in MI towards ‘After’ incorrect frames. Crucially, this bias was significantly smaller for imagining robotic actions compared to human actions. This difference was primarily driven by an increased number of ‘Before’ errors for longer robotic actions, suggesting a perceived slowing down of MI (leading to an attenuated forward bias) when simulating less biologically plausible robotic movements, especially over extended durations. For human actions, video duration did not significantly modulate temporal errors. This finding indicates that the temporal distortion in MI is modulated by the observed agent’s movement characteristics and action duration, challenging simplistic embodied simulation accounts.

**Supplementary Information:**

The online version contains supplementary material available at 10.1007/s00426-026-02259-9.

## Introduction

Motor imagery (MI), the ability to mentally simulate actions without overt movement, is a fundamental cognitive function. This internal simulation is widely believed to rely, at least partially, on the same neural substrates involved in action execution and observation, highlighting the deeply embodied nature of cognition and the tight coupling between perception and action (Decety, 1996; Decety & Grèzes, 1999; Rizzolatti & Craighero, 2004). MI is not merely a passive replay of sensory information but an active process that allows us to anticipate action outcomes, plan future movements, and understand the actions of others. Understanding the temporal dynamics of this internal simulation – how it unfolds in time – is crucial for a comprehensive account of action cognition.

A significant body of research has investigated the temporal characteristics of motor imagery using mental chronometry. These studies typically compare the time taken to mentally simulate an action with the time required to physically execute the same action. A widely reported finding is a strong temporal congruence between imagined and executed movement durations, often referred to as the “isomorphism principle” or “motor imagery chronometry” (Collet et al., 2011; Decety et al., 1989; Guillot & Collet, 2005; Parsons, 1994). While some studies have suggested minor discrepancies, such as imagined movements being slightly faster or slower than executed ones depending on action complexity or load (Dahm & Rieger, 2016; Decety et al., 1989), the overall finding highlights that the internal temporal representation of an action in MI closely mirrors its real-world execution time. However, these studies primarily focus on the total duration of the imagined movement. Less is known about the fine-grained internal temporal unfolding of MI – specifically, whether the subjective experience of the action’s progression maintains a perfectly veridical timeline or exhibits systematic biases or shifts throughout its course, and how factors like action duration and complexity might differentially influence these temporal distortions (Calmels et al., 2006).

A promising framework for understanding the nature of these internal temporal distortions comes from the study of how the brain represents and predicts the time course of moving stimuli. A well-established phenomenon in visual perception is Representational Momentum (RM), the tendency to misremember the final position of a briefly presented moving object as being displaced forward in the direction of its motion (Freyd & Finke, 1984; Hubbard, 2005). RM is often interpreted as reflecting the brain’s predictive mechanisms, anticipating where the object would have gone. While initially studied for simple moving dots or shapes, RM has also been reported for complex dynamic stimuli, including biological motion (Thornton & Hayes, 2004; Zucchini et al., 2023). Crucially, the magnitude of this forward displacement can be modulated by the physical properties of the stimulus (Hubbard, 2017) and the perceived characteristics of the motion (Finke et al., 1986; Nagai et al., 2002).

Given the strong link between action perception, execution, and imagery, a key question is whether similar temporal biases characterize the internal time course of motor imagery. Does mentally simulating an action lead to a temporal representation that is a perfect, time-locked replica of observed or executed action, or does it exhibit its own dynamic properties, potentially including biases akin to RM? To address this gap regarding the fine-grained temporal unfolding and potential biases in MI, the present study investigates the influence of what action is being imagined, particularly actions performed by agents with differing movement characteristics (e.g., biological plausibility and intrinsic duration) like robots and humans, on the temporal dynamics of MI, which is an under-explored area.

As robots become increasingly integrated into human environments, understanding how observing robotic actions influences human action cognition, including MI, is growing in importance (Chaminade & Hodgins, 2006). Prior research on human observation of robotic actions has yielded mixed results regarding the engagement of the human motor system compared to observing human actions, with findings dependent on factors like anthropomorphism and movement naturalness (Gazzola et al., 2007; Hofree et al., 2015; Kilner et al., 2003; Press et al., 2005; Tai et al., 2004). Additionally, behavioral studies comparing human processing and prediction of human and robot actions also reveal differences. For instance, individuals exhibit distinct temporal prediction strategies when observing human versus robotic grasping (Craighero et al., 2008), tend to imitate a robot’s action means rather than its goals unlike human-human interaction (Bao & Cuijpers, 2017), and show modulated visuomotor priming effects, including a reversed pattern for children with autism when observing robots versus humans (Gowen & Poliakoff, 2012; Pierno et al., 2008). Furthermore, the precision of trajectory extrapolation is reduced for movements that violate biological kinematic laws (Pozzo et al., 2006).

Building on these observations regarding action processing and motor simulation, the present study investigates the temporal dynamics of motor imagery specifically, asking whether the specific characteristics of the observed agent’s actions influence how the subsequent mental simulation unfolds in time when performing actions with the same goal (e.g., grasping, pouring, drawing). We employed a temporal judgment task designed to probe the internal time course and accuracy of MI. Participants first observed a complete goal-directed action performed by either a human or a NAO robot (https://us.softbankrobotics.com/nao). These agents were selected because they are known to have distinct kinematic profiles (Flash & Hogan, 1985; Gulletta et al., 2020; Todorov & Jordan, 1998; Viviani & Flash, 1995) and differentially engage sensorimotor systems based on their biological plausibility (Cross et al., 2012; Longo et al., 2008). Notably, inherent biomechanical and control differences mean that the same goal-directed actions often take longer for the robot to execute compared to a human, even when striving for optimal motion. While performing motor imagery of the observed action, participants were interrupted by a stop signal and asked to judge which of two static frames best corresponded to their imagery stop point: the correct one or an alternative frame (either ‘Before’ or ‘After’ the stop point). This task allows us to assess if MI exhibits a temporal bias and if the accuracy of temporal judgment, and thus potentially the magnitude of any bias, is influenced by the type of agent whose action was initially observed.

Furthermore, we explored whether the observer’s own motor system availability, manipulated through arm restraint (free vs. restrained), interacts with agent type to influence MI temporal judgment accuracy, given prior work on arm posture/restraint effects on perception (Alaerts et al., 2009; Urgesi et al., 2006; Zimmermann et al., 2013) and motor imagery (Guilbert et al., 2021; Lorey et al., 2009; Meugnot et al., 2014; Qu et al., 2018; Toussaint & Meugnot, 2013; Vargas et al., 2004).

By addressing these questions, our study aims to provide novel insights into the interplay between observed agent characteristics, observer’s motor state, and the internal temporal unfolding of motor imagery, contributing to a more refined understanding of action simulation and human-robot interaction.

## Materials and methods

### Participants

Twenty-eight right-handed volunteers (15 females, 13 males; Mean age = 23.4 years, SD = 1.83 years) participated in the study.

A priori power analysis was conducted using G*Power 3.1 for a repeated-measures ANOVA design. The analysis assumed a medium effect size (f = 0.30), a significance level of α = 0.05, a desired statistical power of 0.95, an estimated correlation among repeated measures of 0.5, and a nonsphericity correction factor (ε) of 1. The results indicated that a minimum sample size of 17 participants would be required to detect statistically significant effects under these conditions.

Participants were recruited through university announcements and provided written informed consent prior to participation. Inclusion criteria were right-handedness, self-reported normal or corrected-to-normal vision, and no history of neurological or motor disorders. All participants were naive to the purpose of the study.

The study was conducted in accordance with the ethical principles outlined in the Declaration of Helsinki. The experimental protocol was approved by the Ethical Committee of Area Vasta Emilia Centro (Protocol n. 492/2023/Oss/AOUFe).

### Apparatus, stimuli, and procedure

The experiment was conducted in a dedicated, sound-attenuated room to minimize external distractions. Participants were seated comfortably in a chair with armrests, positioned 60 cm from a 27-inch LCD monitor. Stimuli were presented using PsychoPy (version 2023.2.3) running on a Windows 11 PC. The auditory signals were transmitted through the computer speakers at a comfortable listening volume. Responses were collected using a standard QWERTY keyboard.

Visual stimuli consisted of six video clips depicting identical goal-directed actions performed by either a human actor or a NAO robot. The NAO robot, a humanoid platform approximately 58 cm tall developed by SoftBank Robotics, is designed for research in human-robot interaction due to its anthropomorphic form. While programmed to mimic human actions, NAO’s movements are fundamentally different from biological motion. Its motion is often described as ‘jerky’ and exhibits different kinematic properties compared to human actions, typically involving more segmented velocity profiles and greater regularity (i.e., lower trial-to-trial variability and lack of biological noise) due to the constraints of its actuator control and algorithmic implementation (e.g., Flash & Hogan, 1985; Gulletta et al., 2020).

The actions were: grasping a moka pot and pouring coffee into a cup (moka action), grasping a flower and placing it into a vase (flower action), and grasping a marker to draw a line on a sheet of paper (marker action). These three actions were consistently performed by both the human actor and the NAO robot, ensuring that the goal of the action remained constant across agent types. The videos are available in the Supplementary Material (HumanFlower.mp4, HumanMarker.mp4, HumanMoka.mp4, RobotFlower.mp4, RobotMarker.mp4, RobotMoka.mp4).

The videos featuring the robot were obtained by programming it explicitly using the Choreographe (Pot et al., 2009) software suite that allows the user to perform fine-tuning of complex joint or Cartesian motions. For each video, we provided Choreographe with three vectors: the first encodes a sequence of joints, the second a sequence of angular positions for the respective joints, and the third a sequence of time intervals. The angular positions for the joints identify a set of intermediate positions during the action that were determined by trial and error so that NAO performs the desired action. Then we asked Choreographe to interpolate between the intermediate positions to obtain a fluid motion.

Videos were stopped as soon as the action was completed and before the hand started to return to the initial position. Videos were filmed from a third-person perspective, showing the agent (human or robot) performing the action against a neutral background under consistent lighting conditions. The duration of the video clips was slightly longer for the robot (moka action = 5.73 s; flower action = 9.03 s; marker action = 7.27 s) than for the human (moka action = 4.77 s; flower action = 5.36 s; marker action = 4.10 s) agent. Specifically, the range of durations for robotic actions (from 5.73 s to 9.03 s, a range of 3.30 s) was considerably wider than for human actions (from 4.10 s to 5.36 s, a range of 1.26 s). This inherent difference meant that while we controlled for the same goal-directed actions, their natural execution times and variability differed. This difference in duration reflects the inherent kinematic disparities between human biological motion and the programmed movements of the NAO robot, making it challenging to achieve perfectly matched temporal profiles for the same goal-directed actions. These common goal-directed actions were selected for their familiarity and representation of everyday motor skills allowing for direct comparison of motor imagery across agent types for functionally equivalent movements.

Prior to the experimental sessions, participants completed a practice session consisting of 8 trials. The practice session served to familiarize participants with the task, ensuring they understood the instructions and minimizing potential learning effects during the main experiment. The practice trials followed the same structure as the experimental trials.

The experiment comprised two sessions, differing only in the condition of the participant’s dominant upper limb: a ‘free arm’ session and a ‘restrained arm’ session. The order of the sessions (free-restrained vs. restrained-free) was counterbalanced across participants to control for potential order effects. In the ‘restrained arm’ session, participants were instructed to place their dominant hand under their right leg, resting it on the chair seat beneath their thigh. This specific posture, positioning the limb in an unusual and potentially incongruent configuration for the imagined actions, was chosen to modulate the peripheral motor system’s state. This posture served to effectively restrain movement of the dominant arm throughout the session while maintaining a comfortable seated position. Indeed, even subtle changes in arm posture, particularly those considered incompatible with an imagined movement, are known to modulate corticospinal excitability during motor imagery (Vargas et al., 2004), suggesting that this manipulation, though passive, was physiologically grounded to impact the peripheral motor system.

At the end of each experimental session (i.e., ‘free arm’ and ‘restrained arm’ sessions), participants completed a brief questionnaire assessing their subjective experience of motor imagery. They were asked to rate the perceived difficulty of imagining actions performed by the human agent and the robot agent on a 5-point Likert scale (1 = ‘Extremely Difficult’, 5 = ‘Extremely Easy’). This self-report measure aimed to capture participants’ subjective impression of the cognitive effort involved in simulating movements from the different agents, and to explore if this perception varied depending on the availability of their dominant arm.

Each experimental session consisted of 24 trials. At the beginning of each trial, a black fixation cross was presented centrally on a grey background for 3 s. Following the fixation cross, one of the six video clips was randomly selected and displayed. Immediately after video offset, the instruction ‘Close Your Eyes’ appeared on the screen, prompting participants to close their eyes. Two seconds after the ‘Close Your Eyes’ instruction, an auditory ‘start’ signal (440 Hz pure tone, 300 ms duration) was presented, cueing participants to begin motor imagery of the observed action from a first-person perspective. Participants were instructed to imagine performing the action with the same speed, trajectory, and effort as observed in the video. An auditory ‘stop’ signal (523 Hz pure tone, 300 ms duration) was presented at a time point corresponding to 50% of the pre-measured video duration, interrupting the motor imagery process.

Immediately following the ‘stop’ signal, two static frames extracted from the video clip were presented simultaneously side-by-side on the screen. One frame (‘correct frame’) corresponded to the exact video frame at the instant of the ‘stop’ signal (i.e., 50% of the video duration). The other frame (‘incorrect frame’) was selected to be either 25% of the video duration (‘frame before’) or 75% of the video duration (‘frame after’), randomized across trials. The time points for the incorrect probe frames (25% and 75% of video duration) were chosen to create a symmetrical temporal distance from the correct stop frame (50% of video duration), equivalent to 25% of the total action duration. This proportional scaling relative to each video’s overall length was implemented to ensure that the temporal judgment task’s difficulty, in terms of absolute time difference, was relatively consistent across actions, despite variations in their total durations. Choosing time points halfway between the start/end of the action and the stop point provided a balanced temporal window for assessing potential temporal biases both preceding and succeeding the interruption point and was deemed adequate for capturing systematic shifts in motor imagery timing.

On average, the temporal distance separating the correct frame from the incorrect ones (equivalent to 25% of the total action duration) was 1.51 s across all video clips (SD = 0.45 s; see Table 1).

**Table 1:** Temporal characteristics of video stimuli for human and robot actions

agent	human			robot		
action	moka	flower	marker	moka	flower	marker
video duration	4.77	5.36	4.1	5.73	9.03	7.27
correct (50%)	2.39	2.68	2.05	2.87	4.52	3.64
before (25%)	1.19	1.34	1.03	1.43	2.26	1.82
after (75%)	3.58	4.02	3.08	4.30	6.67	5.45
Temp. distance	1.19	1.34	1.03	1.43	2.26	1.82

The table presents the duration (in seconds) for each of the six video clips. It also details the specific time points (in seconds) for: the ‘correct frame’ (at 50% of video duration), the ‘incorrect frame before’ (at 25% of video duration), and the ‘incorrect frame after’ (at 75% of video duration). Furthermore, the temporal distance (equivalent to 25% of video duration) between the incorrect frames and the correct frame is shown

**Fig. 1:**
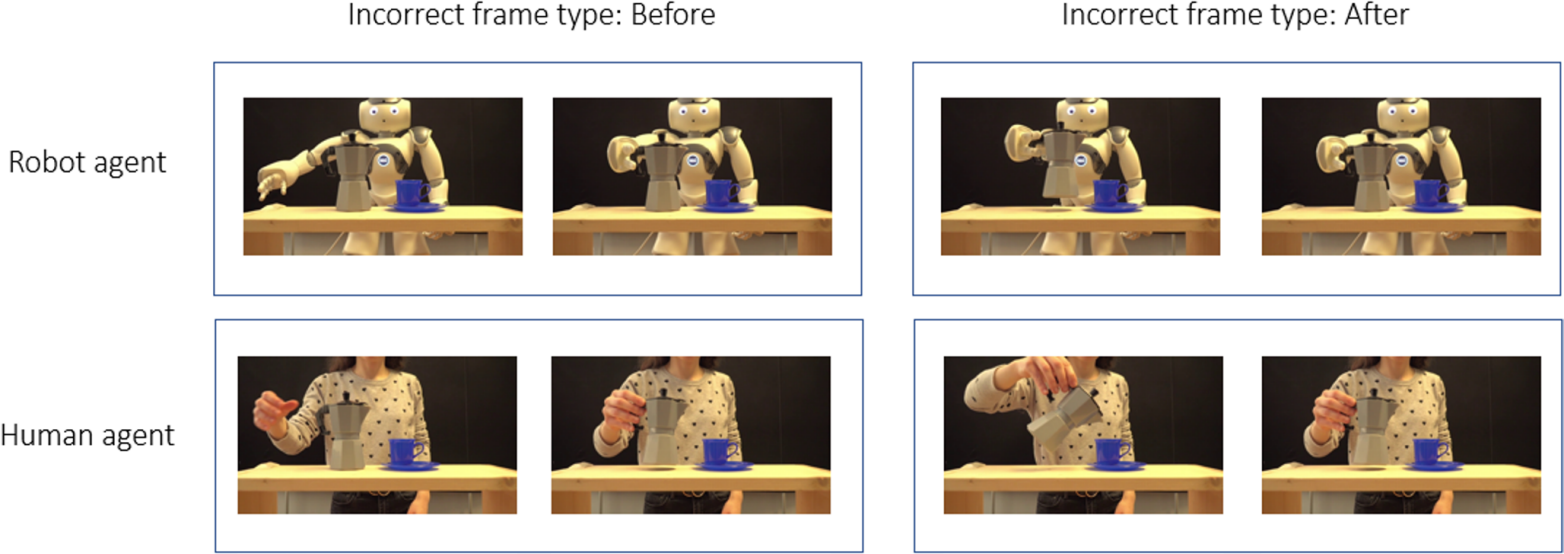
Example of the two static frames extracted from the video clip and presented for the temporal judgment task. As an example, the picture shows frames from the ‘moka action’. The figure illustrates the four experimental conditions (Agent Type: Robot/Human; Incorrect Frame Type: Before/After). Note: In this illustrative figure, the ‘Correct frame’ is shown on the right. During the actual experiment, the position (left/right) of the ‘Correct’ frame was randomized across trials

**Fig. 2:**
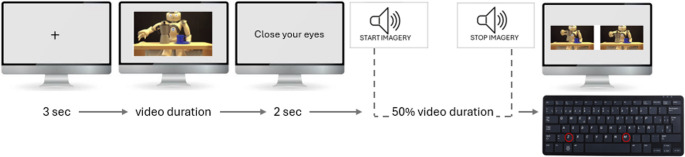
Schematic representation of the experimental procedure. After the fixation cross (3 s) one of the six video clips was displayed. Immediately after video offset, the instruction “Close Eyes” appeared on the screen (2 s). An auditory ‘start’ signal cued participants to begin motor imagery. An auditory ‘stop’ signal was presented at a time point corresponding to 50% of the video duration, interrupting the motor imagery process. Two static frames extracted from the video clip were presented simultaneously side-by-side on the screen. Participants selected the frame (left or right, using the ‘Z’ or ‘M’ key) that they judged to best correspond to the moment their motor imagery of the action was interrupted

The position of the ‘correct frame’ (left or right side of the screen) was also randomized across trials (Fig. 1). The frames lasted until response. Participants were instructed to indicate which of the two frames, left or right, corresponded to the moment when their motor imagery was interrupted by the auditory ‘stop’ signal. Responses were made by pressing with the left hand the ‘Z’ key to select the left frame or the ‘M’ key to select the right frame (Fig. 2). Participants kept their left hand resting on the table next to the keyboard, and typically used their index finger to press the response keys. The left hand was used for responses across all conditions to maintain consistency, particularly given that the ‘restrained arm’ condition always involved the participant’s dominant (right) arm. Participants were encouraged to respond accurately and quickly, although no time limit was imposed. Notably, this temporal judgment task based on the two-alternative forced choice (2-AFC) is based on a paradigm successfully employed in previous research investigating the temporal dynamics of motor imagery, particularly in studies by Galli and colleagues (Galli et al., 2022, 2025), thereby facilitating comparisons with existing literature.

The experiment employed a within-subjects, full factorial design. Each participant completed a total of 48 trials, resulting from the combination of: 2 Arm Conditions (free vs. restrained) × 2 Agent types (human vs. NAO robot) × 3 Action types (Moka vs. Flower vs. Marker) × 2 Incorrect Frame Types (before vs. after) × 2 Correct Frame Positions (left vs. right). The order of trials within each session was randomized for each participant to minimize potential sequence effects, including those related to repeated exposure to specific agent and action combinations or potential carry-over effects between different agent types.

The stimuli presented and the raw data recorded are archived in the research data repository OSF at the link https://osf.io/g84p6/?view_only=054e75aed0ef4f529069ca0252979aa4.

## Data analysis and results

For each participant, for each condition, we calculated the percentage of trials in which an error occurred out of the total number of trials. Errors were defined as trials where the participant incorrectly identified the frame corresponding to the motor imagery interruption. Data from the practice session were excluded from all analyses. Data were analyzed using JASP software (version 0.18.1).

**Fig. 3:**
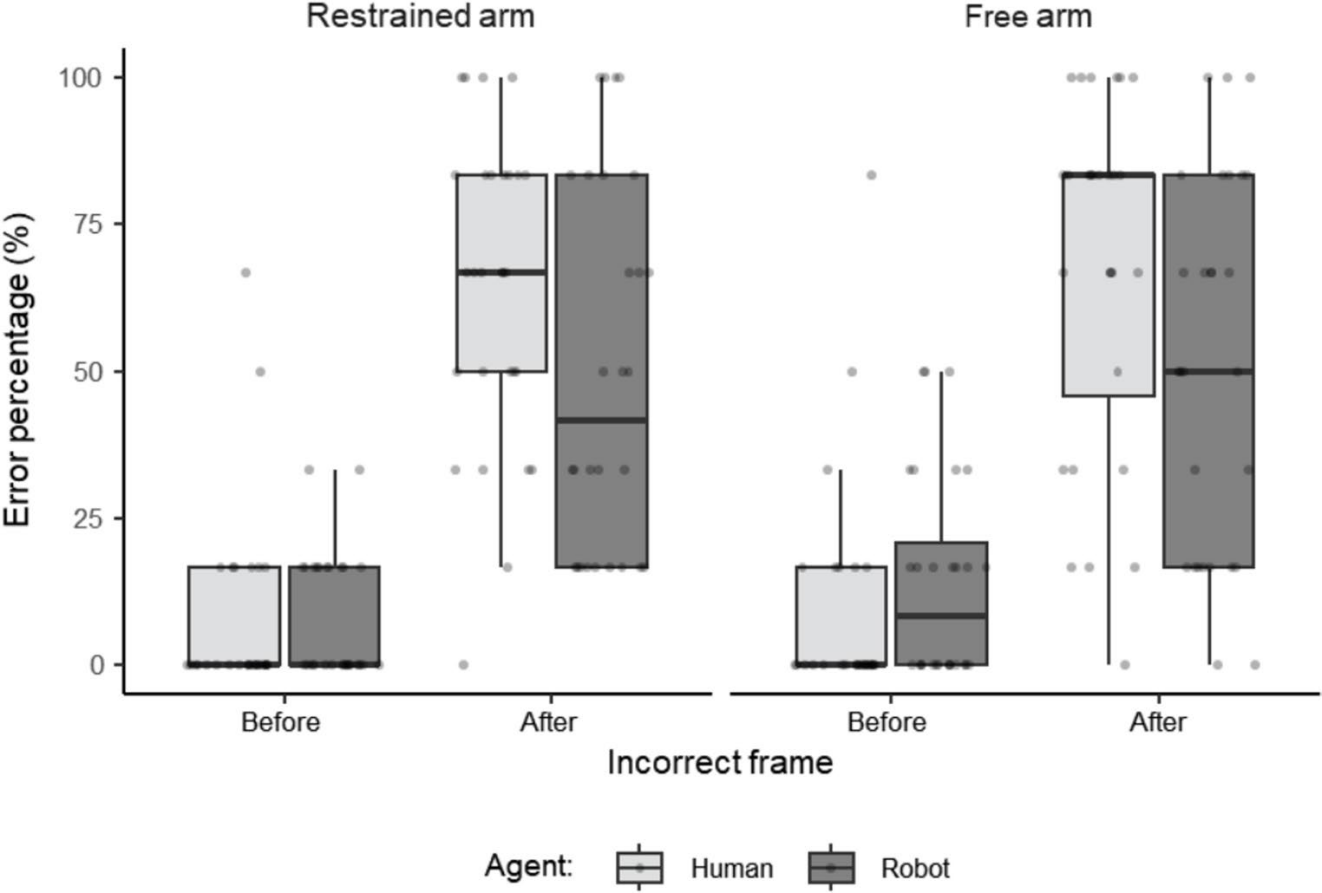
Distribution of error percentage across agent, arm condition, and incorrect frame type. The jittered boxplot illustrates the error percentage for the within-subject factors of Agent (Human vs. Robot), Arm Condition (Free vs. Restrained), and Incorrect Frame Type (Before vs. After)

A repeated-measures ANOVA was conducted with within-subject factors of Agent (Human vs. Robot), Arm Condition (Free vs. Restrained), and Incorrect Frame Type (Before vs. After). Alpha level was set at *p* < .05. Figure 3 displays the data according to the full factorial design.

The analysis revealed a significant main effect of Agent, F(1, 27) = 5.42, *p* = .03, η² = 0.008. Participants exhibited significantly lower error rates when imagining actions performed by the Robot (Mean = 31.0%, SD = 13.1) compared to the Human (Mean = 36.9%, SD = 11.9) agent across all conditions.

There was also a significant main effect of Incorrect Frame Type, F(1, 27) = 96.51, *p* < .001, η² = 0.54. Participants made significantly more errors when the Incorrect Frame Type was After (Mean = 58.0%, SD = 21.2) compared to when it was Before (Mean = 9.8%, SD = 10.5) across all conditions.

The main effect of Arm Condition was not significant, F(1, 27) = 1.11, *p* = .30, η² = 0.002, indicating that restraining the dominant arm did not significantly influence overall error rates.

A significant interaction between Agent and Incorrect Frame Type was observed, F(1, 27) = 8.16, *p* = .008, η² = 0.02. Post-hoc pairwise comparisons with Bonferroni correction further elucidated this interaction. For Before incorrect frames, there was no significant difference in error rates between Robot (Mean = 11.3%, SD = 11.2) and Human (Mean = 8.3%, SD = 15.0) agents. However, for After incorrect frames, participants made significantly fewer errors when imagining actions performed by the Robot (Mean = 50.06%, SD = 25.8) compared to the Human agent (Mean = 65.5%, SD = 23.8), t = 3.08, *p* = .03. This indicates that the effect of Agent on accuracy was dependent on the Incorrect Frame Type.

All other interactions were non-significant (*p* > .05).

**Fig. 4:**
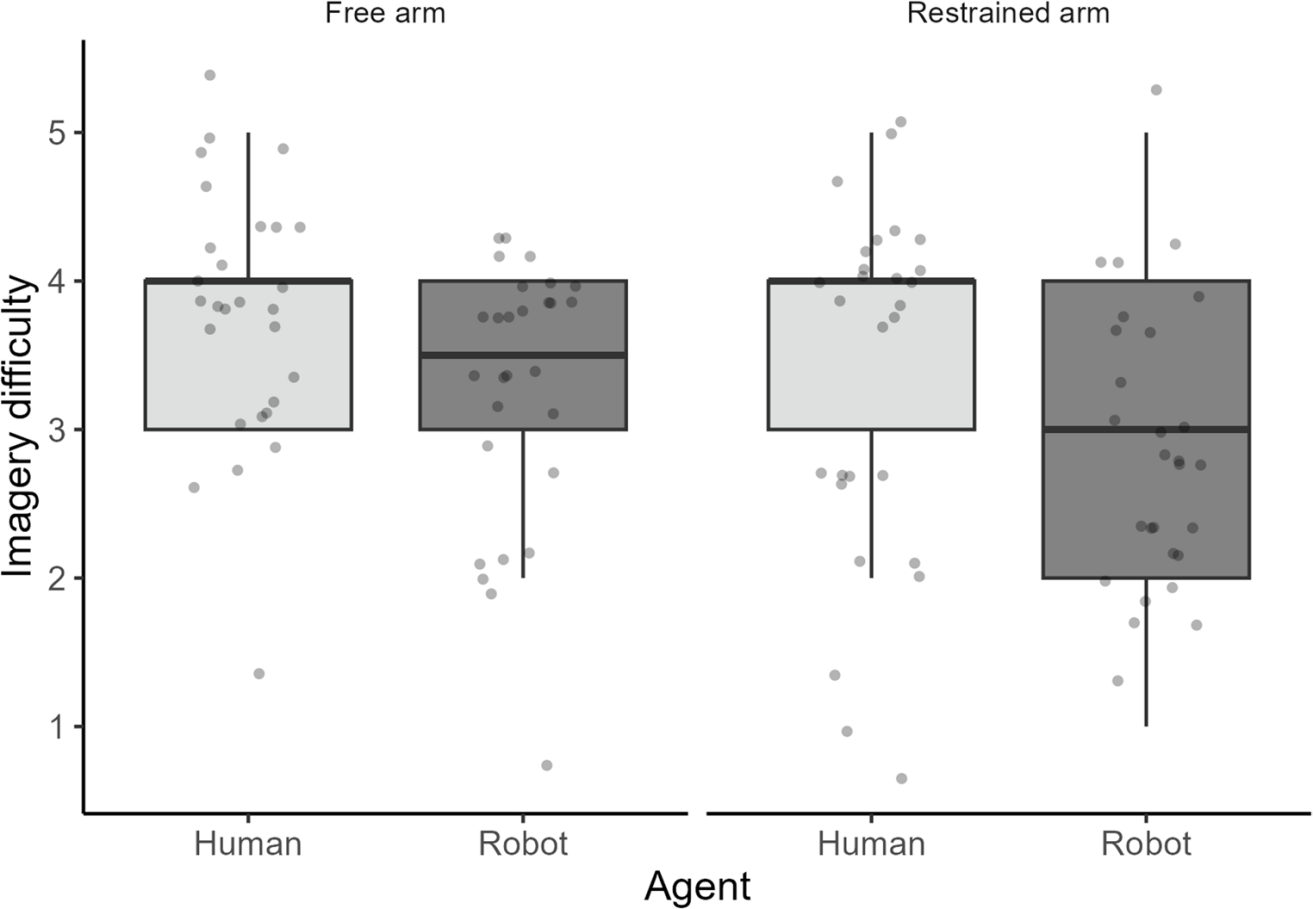
Subjective ratings of imagery difficulty across agent and arm condition. The jittered boxplot illustrates the subjective ratings of imagery difficulty (1 = very difficult, 5 = very easy) for the within-subject factors of Agent (Human vs. Robot) and Arm Condition (Free vs. Restrained)

To assess the perceived difficulty of motor imagery, responses from the post-experiment questionnaire were analyzed using a 2 (Agent: Human vs. Robot) x 2 (Arm Condition: Free vs. Restrained) repeated-measures ANOVA. The dependent variable was the subjective rating of imagery difficulty (on a 1–5 Likert scale, with lower scores indicating greater difficulty). The ANOVA revealed a significant main effect of Agent (F(1, 27) = 8.36, *p* < .01, η² = 0.12). Participants consistently reported significantly greater difficulty imagining actions performed by the robot agent (overall Mean = 3.05, SD = 0.94) compared to the human agent (overall Mean = 3.59, SD = 1.04). A significant main effect of Arm Condition was also observed (F(1, 27) = 4.93, *p* = .04, η² = 0.6). Overall, participants reported greater difficulty imagining actions when their arm was restrained (overall Mean = 3.13, SD = 1.07) compared to when it was free (overall Mean = 3.52, SD = 0.94). Crucially, the interaction between Agent and Arm Condition was not statistically significant (F(1, 27) = 0, *p* = 1.00, η² = 0). This indicates that the perceived difficulty of imagining robot actions was consistently higher than human actions, irrespective of whether the participant’s arm was free or restrained, demonstrating that the differential difficulty of simulating robot versus human actions is robust to the peripheral motor state (Fig. 4).

To address the possibility of participants adopting adaptive strategies over the course of the experiment due to repeated interruptions (e.g., speeding up or slowing down their imagery), we conducted an additional control analysis. We ran a mixed-effects logistic regression including all trials, with Trial Number, Error Type (Before vs. After), and their interaction as fixed effects, and random intercepts for participants. The analysis revealed a strong main effect of Error Type (Estimate = 2.42, SE = 0.30, z = 8.03, *p* < .001), indicating that After errors occurred more frequently than Before errors overall. Neither the main effect of Trial Number (Estimate = − 0.016, SE = 0.010, z = − 1.71, *p* = .09) nor the Trial Number × Error Type interaction (Estimate = 0.012, SE = 0.011, z = 1.04, *p* = .30) reached significance. These results indicate that response patterns remained stable across the session, suggesting that participants did not progressively adapt or accelerate their internal simulation strategy.

Given the inherent disparities between human and robotic motion, which naturally led to longer durations for robotic actions, and crucially, a considerably wider range of durations for robotic actions (from 5.73s to 9.03s, a range of 3.30s) compared to human actions (from 4.10s to 5.36s, a range of 1.26s) (as detailed in Methods), we conducted an additional analysis to explore how action duration specifically modulated the observed temporal biases. Prompted by the significant interaction between Agent and Incorrect Frame Type found in the primary analysis—which indicated that the agent’s influence on accuracy was dependent on the error type—two factorial ANOVAs were performed for ‘Before’ and ‘After’ error percentages. The within-subject factors were ‘Agent’ (Human vs. Robot) and ‘Relative Stimulus Duration’ (3 levels). To create the ‘Relative Stimulus Duration’ factor, the three specific durations for each agent were categorized into ‘Fast,’ ‘Mid,’ and ‘Slow’ based on their relative length within that agent’s repertoire (as detailed in Table 1). Specifically, for human actions, the Marker action (4.10s) was classified as ‘Fast,’ Moka (4.77s) as ‘Mid,’ and Flower (5.36s) as ‘Slow.’ Similarly, for robotic actions, Moka (5.73s) was ‘Fast,’ Marker (7.27s) was ‘Mid,’ and Flower (9.03s) was ‘Slow.’

For ‘After’ frames, the two-way ANOVA revealed a significant main effect of Agent, F(1, 27) = 9.46, *p* = 0.005, η² = 0.07, confirming that participants made significantly fewer ‘After’ errors when imagining actions performed by the Robot compared to the Human agent. A significant main effect of Relative Stimulus Duration was also observed, F(2, 54) = 7.27, *p* = 0.002, η² = 0.04. Bonferroni-corrected post-hoc comparisons (Holm adjusted) for Relative Stimulus Duration indicated a significant decrease in ‘After’ errors from ‘faster’ to ‘slower’ durations (Mean Difference = 6.25, t = 3.44, *p* = 0.006). The comparison between ‘middle’ and ‘slower’ durations was borderline significant (Mean Difference = 4.46, t = 2.39, *p* = 0.05), while no significant difference was found between ‘faster’ and ‘middle’ durations (Mean Difference = 1.79, t = 1.35, *p* = 0.19). The interaction between Agent and Relative Stimulus Duration was not significant, F(2, 54) = 2.24, *p* = 0.12, η² = 0.01. This suggests that the overall tendency for ‘After’ errors to decrease with increasing action duration is consistent across agent types.

**Fig. 5:**
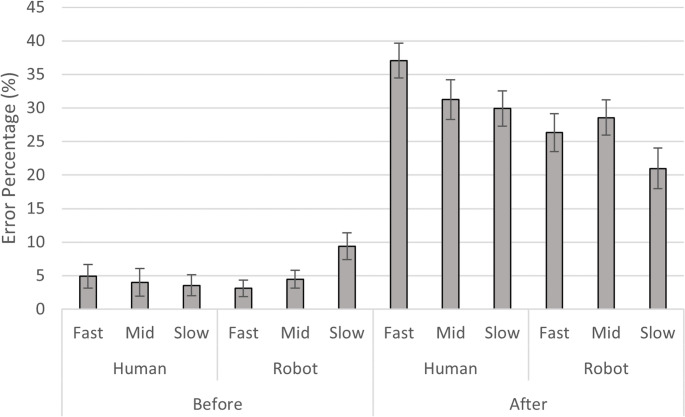
Error percentage distribution across agent, incorrect frame type, and relative stimulus duration. The bar chart illustrates the mean error percentage for the within-subject factors of Agent (Human vs. Robot) and Incorrect Frame Type (‘Before’ vs. ‘After’). For each agent and incorrect frame type, the three bars represent the Relative Stimulus Duration, categorized into ‘Fast,’ ‘Mid,’ and ‘Slow.’ ‘Fast’ corresponds to the shortest action duration within that agent’s repertoire (Human: Marker, 4.10s; Robot: Moka, 5.73s). ‘Mid’ corresponds to the medium duration (Human: Moka, 4.77s; Robot: Marker, 7.27s). ‘Slow’ corresponds to the longest duration (Human: Flower, 5.36s; Robot: Flower, 9.03s). Error bars represent standard errors of the mean (SEM)

For ‘Before’ frames, the two-way ANOVA did not reveal a significant main effect of Agent, F(1, 27) = 0.96, *p* = 0.34, η² = 0.00, nor a significant main effect of Relative Stimulus Duration, F(2, 54) = 1.98, *p* = 0.15, η² = 0.01. However, a significant interaction between Agent and Relative Stimulus Duration was observed, F(2, 54) = 4.10, *p* = 0.02, η² = 0.04. Though Bonferroni-corrected post-hoc comparisons (Holm adjusted) revealed no statistically significant pairwise differences, descriptive statistics (see also Fig. 5) showed that for robot actions, ‘Before’ errors numerically increased with increasing duration (‘faster’: M = 3.13, SD = 6.48; ‘middle’: M = 4.46, SD = 6.98; ‘slower’: M = 9.38, SD = 10.55). In contrast, for human actions, ‘Before’ error percentages remained relatively low and stable across durations (‘faster’: M = 4.91, SD = 9.22; ‘middle’: M = 4.02, SD = 10.79; ‘slower’: M = 3.57, SD = 8.23).

## Discussion

The present study employed a temporal judgment task to investigate the internal time course and accuracy of motor imagery (MI) following the observation of goal-directed actions performed by human and robotic agents. Our primary findings reveal a consistent temporal bias in MI, significantly modulated by the observed agent’s movement characteristics and action duration, while performing the same goal-directed actions and remaining unaffected by the observer’s arm mobility in the short term.

A striking result is the systematic bias towards selecting the ‘After’ incorrect frame over the ‘Correct’ frame when participants’ motor imagery was interrupted. Notably, this finding of an accelerated time course in motor imagery, evidenced by the ‘After’ bias, mirrors recent results obtained using a similar temporal judgment paradigm in studies with children (Galli et al., 2022, 2025). This convergence of results across different populations (adults vs. children) suggests that the observed temporal bias may be a general characteristic of MI, irrespective of the specific observer population. This result indicates that participants consistently judged their imagined actions as having progressed temporally beyond the actual point of the stop signal interruption. Further analysis confirmed that these response patterns remained stable across trials, with no evidence of systematic strategic adaptation over time.

This systematic forward temporal bias in MI exhibits conceptual parallels with Representational Momentum (RM), a well-established phenomenon in visual perception where the remembered final position of a briefly presented moving object is displaced forward in the direction of its motion (Freyd & Finke, 1984; Hubbard, 2005; see also Hubbard, 2017 for a general theory of momentum-like effects). However, it is important to distinguish that our task involves judging an interruption point rather than a final position, thus the underlying mechanisms may differ. This forward temporal bias in MI suggests an anticipatory and dynamic unfolding of the internal simulation, where the mental action progresses slightly ahead of the objective timeline. In our data, this is clearly evidenced by the high rate of ‘After’ errors compared to the very low rate of ‘Before’ errors across all conditions. This could manifest as either a perceived accelerated speed of motor imagery or a momentum-like continuation of the simulation even after the stop signal, driven by internal predictive mechanisms, thereby distorting the subjective experience of the exact interruption point.

Crucially, our finding that the magnitude of this forward bias is significantly smaller for imagining robotic actions compared to human actions demonstrates that this temporal bias is not a fixed property of MI but is dynamically influenced by the characteristics of the observed action. A more detailed analysis revealed that this effect is closely related to the duration of the observed actions. Specifically, for ‘After’ errors, there was an overall tendency to decrease with increasing action duration, consistent across agent types, and no significant interaction between Agent Type and Relative Stimulus Duration was observed. However, it is also important to consider an objective aspect of the task that might have contributed to the general decrease in ‘After’ errors with increasing action duration. Given the proportionally scaled temporal distance between the ‘Correct’ and ‘After’ frames, the absolute time difference between these frames was larger for longer videos (as detailed in Table 1). This increased objective discriminability for longer actions could have made the task objectively easier, thereby contributing to the observed reduction in ‘After’ errors across all agent types.

In contrast, for ‘Before’ errors, a significant interaction between Agent Type and Relative Stimulus Duration was observed. This distinct pattern (as visually depicted in Fig. 5) highlights a differential temporal dynamic where for robotic actions ‘Before’ errors numerically increased with increasing duration. This may suggest that the internal simulation of longer robotic movements, which participants rated as more difficult, leads to a more conservative temporal judgment or a perceived slowing of the mental simulation process, thereby resulting in an attenuation of the inherent forward temporal bias rather than an outright lagging behind the objective action. This pattern, notably absent for human actions, indicates a differential sensitivity to action duration depending on the agent type.

A critical consideration when interpreting these differential effects is the inherent difference in the range of durations presented for human versus robotic actions. As detailed in the Methods and Table 1, robotic actions, due to their kinematic characteristics, naturally exhibited a considerably wider range of durations (3.30s) compared to human actions (1.26s). While this wider range for robot videos could, in principle, provide greater statistical power to detect duration-dependent effects, the absence of a significant duration effect for human actions, even within their tested range, is noteworthy. In contrast, the significant reduction in ‘After’ errors for longer robotic actions, combined with the observed numerical trend towards more ‘Before’ errors and participants’ reported higher difficulty in imagining these movements, suggests a genuine difference in the cognitive processing demands of non-biological actions, especially as they become more protracted. Therefore, while our findings strongly indicate a modulation of MI temporal dynamics by agent type and action duration, we cannot definitively disentangle the effect of agent type from the full range of possible durations without a study specifically designed to match or systematically vary duration ranges across agent types. Future research should explicitly address this by either matching duration ranges or exploring a broader, systematically varied set of durations for both agents to further elucidate these interactions.

To reconcile the observed anticipatory temporal bias with the slowing for robotic actions, we propose that the intrinsic forward drive is actively modulated by the perceived ‘ease’ or ‘efficiency’ of the internal simulation. For human actions, where the simulation proceeds smoothly regardless of duration, this forward bias manifests more strongly. In contrast, the less biologically plausible movement characteristics and longer durations of robotic movements, which also presented a wider range of temporal variation in our stimuli, appear to demand a higher cognitive load for internal simulation. This increased cognitive demand is strongly supported by participants’ subjective reports from a post-experiment questionnaire, where imagining robot actions was rated as significantly more difficult than imagining human actions, and this differential difficulty persisted regardless of arm condition. This higher cognitive load in simulating robotic actions effectively slows down the perceived pace of motor imagery (leading to a numerical increase in ‘Before’ errors) and, consequently, attenuates the overall expression of the ‘After’ bias. Thus, the reduced ‘After’ errors for robotic actions do not signify an absence of the forward bias tendency, but rather its attenuation due to a concomitant slowing of the internal simulation process, reflecting adaptive adjustments in simulation speed (Flash & Hogan, 1985; Gulletta et al., 2020; Viviani & Flash, 1995). These findings are further supported by a control analysis demonstrating the stability of response patterns throughout the experiment, suggesting that the observed modulation of MI is not attributable to progressive participant strategies. This suggests a complex interplay where the biological congruence and temporal extent of an action influence the speed of the internal simulation, particularly for non-biological or lengthy actions, rather than just the magnitude of the anticipatory overshoot.

To further contextualize our findings, it is important to distinguish our temporal judgment task, which probes the subjective experience of an interruption point during ongoing imagery, from traditional mental chronometry studies that typically compare the total duration of imagined actions with their executed counterparts (e.g., Collet et al., 2011; Parsons, 1994). Our investigated actions – grasping, pouring, and drawing – are common, everyday motor skills. In mental chronometry, such actions often show a strong temporal congruence (isomorphism) between imagined and executed durations, or sometimes a slight acceleration of imagery, particularly for less complex movements (Decety et al., 1989; Guillot & Collet, 2005). Our consistent forward temporal bias in MI can be seen as a fine-grained manifestation of the inherent dynamic and anticipatory nature of motor imagery. This internal drive to project the action slightly ahead of the objective timeline is consistent with observations where MI can be faster than execution, reflecting an efficient internal simulation process. When MI is relatively smooth and efficient, as often assumed for human-like, biologically plausible actions, this anticipatory bias manifests more strongly. Conversely, when MI becomes more cognitively demanding, as evidenced by the increased difficulty and ‘Before’ errors for robotic actions, this forward drive appears to be actively modulated and attenuated. This adaptive slowing of the internal simulation aligns with findings from chronometry studies where more complex or difficult-to-imagine actions can lead to longer MI durations (Dahm & Rieger, 2016). Therefore, anticipatory temporal biases in motor imagery are likely to occur whenever the internal simulation is driven by predictive mechanisms, particularly for fluid, biologically plausible actions that permit efficient internal processing. These biases would be attenuated or modified when cognitive load is high, biological plausibility is low, or when the action is protracted and challenging to simulate.

These findings reveal that temporal bias in MI is not a fixed property but is dynamically modulated by action characteristics—such as biological plausibility and duration—challenging simplistic accounts of motor imagery (e.g., early interpretations emphasizing strict isomorphism, Parsons, 1994; see also Collet et al., 2011). In line with dynamic embodied cognition accounts (Barsalou, 2008; Craighero, 2024; Gallese & Lakoff, 2005; Varela et al., 2016), the present results suggest that mentally simulating an action is grounded in sensorimotor knowledge that leverages observed kinematics to adapt the pace of internal simulations (Flash & Hogan, 1985; Gulletta et al., 2020; Viviani & Flash, 1995). This flexibility is a hallmark of dynamic embodied systems, potentially preventing excessive temporal overshoots or lags (e.g., Kilner et al., 2007; Wolpert et al., 2003; Wolpert & Ghahramani, 2000). The sensitivity of these adaptive simulation processes to the characteristics of observed agents, including robots, is consistent with their flexible tuning based on environmental input (Craighero et al., 2008; Gazzola et al., 2007; Gowen & Poliakoff, 2012). This aligns with a broader body of literature on action observation, which demonstrates that the motor system dynamically adjusts its internal simulations based on the biological congruence and kinematic properties of observed movements, distinguishing between human, robotic, or even biomechanically impossible actions (Costantini et al., 2005; Cross et al., 2012; Longo et al., 2008). These findings highlight that our internal models are finely tuned to the nature of the observed agent, influencing how subsequent motor imagery unfolds.

Furthermore, while models like the ‘efferent delay subtraction’ (Jeannerod, 2001) predict MI might be faster than execution, they do not readily explain our consistent forward temporal bias (‘After’ frame selection) relative to the observed action’s timeline. Our data show an overshoot, suggesting an anticipatory or goal-oriented process leading to temporal compression relative to observation and a forward projection. The modulation of this bias by agent type further supports that the simulation is dynamically influenced by observed movement characteristics, not just a fixed internal mechanism like efferent delay. Our findings resonate with studies using occluder paradigms in action observation, which also explore the timing and representational mechanisms of ongoing action simulations (Sparenberg et al., 2012; Springer et al., 2013). These studies consistently show that the brain actively predicts and simulates the time course of occluded actions, a process that shares conceptual underpinnings with our investigation into MI’s temporal unfolding.

Interestingly, this pattern of modulation, where less biologically plausible robot actions, especially longer ones, lead to an attenuation of the internal simulation speed (relative to the acceleration observed for human actions), manifested as increased ‘before’ errors and a reduced ‘After’ bias, offers a nuanced perspective compared to perceptual Representational Momentum (RM). In RM, less ‘rich’ stimuli sometimes elicit larger forward shifts (Zucchini et al., 2023). Here, in MI, it appears that the cognitive system actively adjusts the simulation speed in response to the characteristics of the observed agent and action. Rather than a simple forward projection, MI involves a more sophisticated adaptation: for movements perceived as more challenging or less natural (like the robot’s), a perception directly confirmed by participants’ higher reported difficulty in imagining robot actions, especially when extended in time, the internal simulation might slow down to maintain accuracy or manage cognitive load. This suggests that factors like the biological congruence of the movement and its duration, and the resultant ease of internal simulation, play a more prominent role in modulating MI temporal biases. The interplay between the inherent forward momentum and the adaptive slowing of simulation, particularly for challenging stimuli like protracted robot actions, defines this distinction. This differs from solely relying on “biological richness” (Zucchini et al., 2023) or direct predictability inferences, suggesting distinct mechanisms underlying temporal distortions in motor imagery versus purely visual perception.

The manipulation of the observer’s dominant arm mobility (free vs. restrained) did not significantly affect temporal judgment accuracy, nor did it interact with agent type or incorrect frame type. This null finding, alongside the non-significant interaction between Agent and Arm Condition on perceived imagery difficulty, suggests that, in this temporal judgment task focusing on the internal unfolding of imagery, MI is primarily driven by central sensorimotor representations. The short-term physical constraint of the peripheral limb, despite being known to modulate corticospinal excitability (Vargas et al., 2004), appears to have a limited impact on the temporal dynamics of mentally simulated actions, and on the differential subjective experience of difficulty between agents, at least as measured by this paradigm. This aligns with theories emphasizing the central, rather than exclusively peripheral, basis of motor imagery (Jeannerod, 2001).

Several limitations warrant consideration. First, the use of a limited set of goal-directed actions performed by a single robotic agent model may restrict the generalizability of our conclusions. Future research should explore a broader range of actions, and agents with varying degrees of anthropomorphism and kinematic profiles, specifically investigating how variations in action duration within each agent type might further modulate the speed and direction of the temporal bias in motor imagery. Second, while we specifically conducted a control analysis to investigate potential participant strategies, which did not reveal systematic changes over time, future studies could still include more detailed subjective reports on imagery vividness, modality, and consistency of perspective (first-person vs. third-person), especially for non-biological agents.

Furthermore, while the two-alternative forced choice (2-AFC) temporal judgment task employed in this study was selected to facilitate comparisons with existing literature (Galli et al., 2022, 2025) this format inherently limits the ability to precisely quantify the full magnitude or exact temporal location of this bias. Specifically, in trials presenting a ‘Correct’ frame alongside a ‘Before’ frame, participants exhibiting a forward temporal bias might select the ‘Correct’ frame simply because it represents the temporally closest, or “less incorrect,” option. Consequently, using a more graded response method, such as a three-alternative forced choice task (‘Before’, ‘Correct’, ‘After’ options presented simultaneously) or a continuous response scale, similar to those employed in occluder paradigms for action observation (Graf et al., 2007; Prinz & Rapinett, 2008), could potentially provide richer data on the precise magnitude of the temporal shift and the speed of internal simulation across conditions in future research. Finally, while our arm restraint manipulation provided physiologically grounded modulation of the peripheral motor system, future investigations could explore even stronger manipulations, such as active concurrent motor tasks, to further investigate the boundaries of its influence on motor imagery temporal dynamics.

## Conclusion

In conclusion, this study provides evidence demonstrating that motor imagery exhibits a consistent forward temporal bias. This bias suggests that internal simulation drives the representation slightly forward beyond the objective stop point. The novel finding is that this bias in MI is not static but dynamically modulated by the characteristics of the observed agent, specifically through the interplay between biological plausibility and action duration, which implicitly reflect differences in underlying kinematics. Imagining human actions leads to a larger ‘After’ temporal shift whereas imagining robotic actions, particularly longer ones, induces a reduction in the internal simulation speed compared to human actions. This attenuation of the forward projection pace, despite the reported higher difficulty, paradoxically contributes to a reduced overall ‘After’ bias for robot actions, reflecting a more conservative temporal judgment. The temporal dynamics of MI, as assessed here, appear largely independent of the observer’s acute peripheral motor state. These findings contribute to a more refined understanding of action simulation, highlighting that MI is a dynamic, anticipatory process influenced by the biological congruence and temporal properties of the observed movement.

## Supplementary Information

Below is the link to the electronic supplementary material.

Supplementary Material 1 (MP4 4.04 MB)

Supplementary Material 2 (MP4 4.50 MB)

Supplementary Material 3 (MP4 5.27 MB)

Supplementary Material 4 (MP4 8.96 MB)

Supplementary Material 5 (MP4 11.4 MB)

Supplementary Material 6 (MP4 14.2 MB)

## Data Availability

The stimuli presented and the raw data recorded are archived in the research data repository OSF at the link https://.osf.io/g84p6/?view\_only=054e75aed0ef4f529069ca0252979aa4.

## References

[CR1] Alaerts, K., Heremans, E., Swinnen, S. P., & Wenderoth, N. (2009). How are observed actions mapped to the observer’s motor system? Influence of posture and perspective. *Neuropsychologia*, *47*(2), 415–422. 10.1016/j.neuropsychologia.2008.09.012 18926836 10.1016/j.neuropsychologia.2008.09.012

[CR2] Barsalou, L. W. (2008). Grounded cognition. *Annual Review of Psychology*, *59*. 10.1146/annurev.psych.59.103006.093639 10.1146/annurev.psych.59.103006.093639 17705682

[CR3] Calmels, C., Holmes, P., Lopez, E., & Naman, V. (2006). Chronometric comparison of actual and imaged complex movement patterns. *Journal of Motor Behavior*, *38*(5), 339–348. 10.3200/JMBR.38.5.339-348 16968679 10.3200/JMBR.38.5.339-348

[CR4] Chaminade, T., & Hodgins, J. K. (2006). Artificial agents in social cognitive sciences. *Interaction Studies Social Behaviour and Communication in Biological and Artificial Systems*, *7*(3), 347–353. 10.1075/is.7.3.07cha

[CR5] Collet, C., Guillot, A., Lebon, F., MacIntyre, T., & Moran, A. (2011). Measuring motor imagery using Psychometric, Behavioral, and Psychophysiological tools. *Exercise and Sport Sciences Reviews*, *39*(2), 85–92. 10.1097/JES.0b013e31820ac5e0 21206282 10.1097/JES.0b013e31820ac5e0

[CR6] Costantini, M., Galati, G., Ferretti, A., Caulo, M., Tartaro, A., Romani, G. L., & Aglioti, S. M. (2005). Neural systems underlying observation of humanly impossible movements: An fMRI study. *Cerebral Cortex*, *15*(11), 1761–1767. 10.1093/cercor/bhi053 15728741 10.1093/cercor/bhi053

[CR7] Craighero, L. (2024). An embodied approach to fetal and newborn perceptual and sensorimotor development. *Brain and Cognition*, *179*, 106184. 10.1016/j.bandc.2024.106184 38843762 10.1016/j.bandc.2024.106184

[CR8] Craighero, L., Bonetti, F., Massarenti, L., Canto, R., Fabbri Destro, M., & Fadiga, L. (2008). Temporal prediction of touch instant during observation of human and robot grasping. *Brain Research Bulletin*, *75*(6), 770–774. 10.1016/j.brainresbull.2008.01.014 18394523 10.1016/j.brainresbull.2008.01.014

[CR9] Cross, E. S., Liepelt, R., de Hamilton, C., Parkinson, A. F., Ramsey, J., Stadler, R., W., & Prinz, W. (2012). Robotic movement preferentially engages the action observation network. *Human Brain Mapping*, *33*(9), 2238–2254. 10.1002/hbm.21361 21898675 10.1002/hbm.21361 PMC6870135

[CR10] Dahm, S. F., & Rieger, M. (2016). Is there symmetry in motor imagery? Exploring different versions of the mental chronometry paradigm. *Attention Perception & Psychophysics*, *78*(6), 1794–1805. 10.3758/s13414-016-1112-9 10.3758/s13414-016-1112-9 PMC4972863 27173486

[CR11] Decety, J. (1996). The neurophysiological basis of motor imagery. *Behavioural Brain Research*, *77*(1–2), 45–52. 10.1016/0166-4328(95)00225-1 8762158 10.1016/0166-4328(95)00225-1

[CR12] Decety, J., & Grèzes, J. (1999). Neural mechanisms subserving the perception of human actions. *Trends in Cognitive Sciences*, *3*(5), 172–178. 10.1016/S1364-6613(99)01312-1 10322473 10.1016/s1364-6613(99)01312-1

[CR13] Decety, J., Jeannerod, M., & Prablanc, C. (1989). The timing of mentally represented actions. *Behavioural Brain Research*, *34*(1–2), 35–42. 10.1016/S0166-4328(89)80088-9 2765170 10.1016/s0166-4328(89)80088-9

[CR14] Finke, R. A., Freyd, J. J., & Shyi, G. C. (1986). Implied velocity and acceleration induce transformations of visual memory. *Journal of Experimental Psychology: General*, *115*(2), 175–188. 10.1037/0096-3445.115.2.175 2940315 10.1037//0096-3445.115.2.175

[CR15] Flash, T., & Hogan, N. (1985). The coordination of arm movements: An experimentally confirmed mathematical model. *The Journal of Neuroscience*, *5*(7), 1688–1703. 10.1523/JNEUROSCI.05-07-01688.1985 4020415 10.1523/JNEUROSCI.05-07-01688.1985 PMC6565116

[CR16] Freyd, J. J., & Finke, R. A. (1984). Representational momentum. *Journal of Experimental Psychology: Learning Memory and Cognition*, *10*(1), 126–132. 10.1037/0278-7393.10.1.126

[CR17] Gallese, V., & Lakoff, G. (2005). The brain’s concepts: The role of the sensory-motor system in conceptual knowledge. *Cognitive Neuropsychology*, *22*(3–4), 455–479. 10.1080/02643290442000310 21038261 10.1080/02643290442000310

[CR18] Galli, J., Garofalo, G., Brunetti, S., Loi, E., Portesi, M., Pelizzari, G., Rossi, A., Fazzi, E., & Buccino, G. (2022). Children with cerebral palsy can imagine actions like their normally developed peers. *Frontiers in Neurology*, *13*. 10.3389/fneur.2022.951152 10.3389/fneur.2022.951152 PMC9488128 36147045

[CR19] Galli, J., Dusi, L., Garofalo, G., Brizzi, A., Gritti, M., Polo, F., Fazzi, E., & Buccino, G. (2025). Children with autistic spectrum disorder can imagine actions— what can this reveal about the Broken Mirror Hypothesis? *Frontiers in Neurology*, *16*. 10.3389/fneur.2025.1490445 10.3389/fneur.2025.1490445 PMC11798804 39916947

[CR20] Gazzola, V., Rizzolatti, G., Wicker, B., & Keysers, C. (2007). The anthropomorphic brain: The mirror neuron system responds to human and robotic actions. *Neuroimage*, *35*(4), 1674–1684. 10.1016/j.neuroimage.2007.02.003 17395490 10.1016/j.neuroimage.2007.02.003

[CR21] Gowen, E., & Poliakoff, E. (2012). How does visuomotor priming differ for biological and non-biological stimuli? A review of the evidence. *Psychological Research Psychologische Forschung*, *76*(4), 407–420. 10.1007/s00426-011-0389-5 22302411 10.1007/s00426-011-0389-5

[CR22] Graf, M., Reitzner, B., Corves, C., Casile, A., Giese, M., & Prinz, W. (2007). Predicting point-light actions in real-time. *Neuroimage*, *36*, T22–T32. 10.1016/j.neuroimage.2007.03.017 17499167 10.1016/j.neuroimage.2007.03.017

[CR23] Guilbert, J., Fernandez, J., Molina, M., Morin, M. F., & Alamargot, D. (2021). Imagining handwriting movements in a usual or unusual position: Effect of posture congruency on visual and kinesthetic motor imagery. *Psychological Research Psychologische Forschung*, *85*(6), 2237–2247. 10.1007/s00426-020-01399-w 32743730 10.1007/s00426-020-01399-w

[CR24] Guillot, A., & Collet, C. (2005). Duration of mentally simulated movement: A review. *Journal of Motor Behavior*, *37*(1), 10–20. 10.3200/JMBR.37.1.10-20 15642689 10.3200/JMBR.37.1.10-20

[CR25] Gulletta, G., Erlhagen, W., & Bicho, E. (2020). Human-Like arm motion generation: A review. *Robotics*, *9*(4), 102. 10.3390/robotics9040102

[CR26] Hofree, G., Urgen, B. A., Winkielman, P., & Saygin, A. P. (2015). Observation And imitation of actions performed by humans, Androids, And robots: An EMG study. *Frontiers in Human Neuroscience*, *9*. 10.3389/fnhum.2015.00364 10.3389/fnhum.2015.00364 PMC4473002 26150782

[CR27] Hubbard, T. L. (2005). Representational momentum and related displacements in Spatial memory: A review of the findings. *Psychonomic Bulletin & Review*, *12*(5), 822–851. 10.3758/BF03196775 16524000 10.3758/bf03196775

[CR28] Hubbard, T. L. (2017). Toward a general theory of momentum-like effects. *Behavioural Processes*, *141*, 50–66. 10.1016/j.beproc.2017.02.019 10.1016/j.beproc.2017.02.019 28257789

[CR29] Jeannerod, M. (2001). Neural simulation of action: A unifying mechanism for motor cognition. *Neuroimage*, *14*(1), S103–S109. 10.1006/nimg.2001.0832 11373140 10.1006/nimg.2001.0832

[CR30] Kilner, J. M., Paulignan, Y., & Blakemore, S. J. (2003). An interference effect of observed biological movement on action. *Current Biology*, *13*(6), 522–525. 10.1016/S0960-9822(03)00165-9 12646137 10.1016/s0960-9822(03)00165-9

[CR31] Kilner, J. M., Friston, K. J., & Frith, C. D. (2007). Predictive coding: An account of the mirror neuron system. In *Cognitive Processing* (Vol. 8, Number 3, pp. 159–166). 10.1007/s10339-007-0170-2 10.1007/s10339-007-0170-2 PMC2649419 17429704

[CR32] Longo, M. R., Kosobud, A., & Bertenthal, B. I. (2008). Automatic imitation of biomechanically possible and impossible actions: Effects of priming movements versus goals. *Journal of Experimental Psychology: Human Perception and Performance*, *34*(2), 489–501. 10.1037/0096-1523.34.2.489 18377184 10.1037/0096-1523.34.2.489

[CR33] Lorey, B., Bischoff, M., Pilgramm, S., Stark, R., Munzert, J., & Zentgraf, K. (2009). The embodied nature of motor imagery: The influence of posture and perspective. *Experimental Brain Research*, *194*(2), 233–243. 10.1007/s00221-008-1693-1 19139856 10.1007/s00221-008-1693-1

[CR34] Meugnot, A., Almecija, Y., & Toussaint, L. (2014). The embodied nature of motor imagery processes highlighted by Short-Term limb immobilization. *Experimental Psychology*, *61*(3), 180–186. 10.1027/1618-3169/a000237 24149241 10.1027/1618-3169/a000237

[CR35] Nagai, M., Kazai, K., & Yagi, A. (2002). Larger forward memory displacement in the direction of gravity. *Visual Cognition*, *9*(1–2), 28–40. 10.1080/13506280143000304

[CR36] Parsons, L. M. (1994). Temporal and kinematic properties of motor behavior reflected in mentally simulated action. *Journal of Experimental Psychology: Human Perception and Performance*, *20*(4), 709–730. 10.1037/0096-1523.20.4.709 8083630 10.1037//0096-1523.20.4.709

[CR37] Pierno, A. C., Mari, M., Lusher, D., & Castiello, U. (2008). Robotic movement elicits visuomotor priming in children with autism. *Neuropsychologia, 46*(2), 448–454. 10.1016/j.neuropsychologia.2007.08.020 10.1016/j.neuropsychologia.2007.08.020 17920641

[CR38] Pot, E., Monceaux, J., Gelin, R., & Maisonnier, B. (2009). Choregraphe: A graphical tool for humanoid robot programming. *RO-MAN 2009 - The 18th IEEE International Symposium on Robot and Human Interactive Communication*, 46–51. 10.1109/ROMAN.2009.5326209

[CR39] Pozzo, T., Papaxanthis, C., Petit, J. L., Schweighofer, N., & Stucchi, N. (2006). Kinematic features of movement tunes perception and action coupling. Behavioural Brain Research, 169(1), 75–82. 10.1016/j.bbr.2005.12.005 10.1016/j.bbr.2005.12.005 16430976

[CR40] Press, C., Bird, G., Flach, R., & Heyes, C. (2005). Robotic movement elicits automatic imitation. *Cognitive Brain Research*, *25*(3), 632–640. 10.1016/j.cogbrainres.2005.08.020 16344220 10.1016/j.cogbrainres.2005.08.020

[CR41] Prinz, W., & Rapinett, G. (2008). Filling the gap: Dynamic representation of occluded action. In F. Morganti, A. Carassa, & G. Riva (Eds.), *Enacting intersubjectivity: A cognitive and social perspective on the study of interactions* (pp. 223–236). IOS.

[CR42] Qu, F., Wang, J., Zhong, Y., & Ye, H. (2018). Postural effects on the mental rotation of Body-Related pictures: An fMRI study. *Frontiers in Psychology*, *9*. 10.3389/fpsyg.2018.00720 10.3389/fpsyg.2018.00720 PMC5975102 29875713

[CR43] Rizzolatti, G., & Craighero, L. (2004). The mirror-neuron system. *Annual Review of Neuroscience*, *27*(1), 169–192. 10.1146/annurev.neuro.27.070203.144230 15217330 10.1146/annurev.neuro.27.070203.144230

[CR44] Sparenberg, P., Springer, A., & Prinz, W. (2012). Predicting others’ actions: Evidence for a constant time delay in action simulation. *Psychological Research Psychologische Forschung*, *76*(1), 41–49. 10.1007/s00426-011-0321-z 21365343 10.1007/s00426-011-0321-z

[CR45] Springer, A., Parkinson, J., & Prinz, W. (2013). Action simulation: Time course and representational mechanisms. *Frontiers in Psychology*, *4*. 10.3389/fpsyg.2013.00387 10.3389/fpsyg.2013.00387 PMC3701141 23847563

[CR46] Tai, Y. F., Scherfler, C., Brooks, D. J., Sawamoto, N., & Castiello, U. (2004). The human premotor cortex is mirror only for biological actions. *Current Biology*, *14*(2), 117–120. 10.1016/j.cub.2004.01.005 14738732 10.1016/j.cub.2004.01.005

[CR47] Thornton, I., & Hayes, A. (2004). Anticipating action in complex scenes. *Visual Cognition*, *11*(2–3), 341–370. 10.1080/13506280344000374

[CR48] Todorov, E., & Jordan, M. I. (1998). Smoothness maximization along a predefined path accurately predicts the speed profiles of complex arm movements. *Journal of Neurophysiology*, *80*(2), 696–714. 10.1152/jn.1998.80.2.696 9705462 10.1152/jn.1998.80.2.696

[CR49] Toussaint, L., & Meugnot, A. (2013). Short-term limb immobilization affects cognitive motor processes. *Journal of Experimental Psychology: Learning Memory and Cognition*, *39*(2), 623–632. 10.1037/a0028942 22686843 10.1037/a0028942

[CR50] Urgesi, C., Candidi, M., Fabbro, F., Romani, M., & Aglioti, S. M. (2006). Motor facilitation during action observation: Topographic mapping of the target muscle and influence of the onlooker’s posture. *European Journal of Neuroscience*, *23*(9), 2522–2530. 10.1111/j.1460-9568.2006.04772.x 16706859 10.1111/j.1460-9568.2006.04772.x

[CR51] Varela, F. J., Thompson, E., Rosch, E., & Kabat-Zinn, J. (2016). The embodied mind: Cognitive science and human experience. In *The Embodied Mind: Cognitive Science and Human Experience*. 10.29173/cmplct8718

[CR52] Vargas, C. D., Olivier, E., Craighero, L., Fadiga, L., Duhamel, J. R., & Sirigu, A. (2004). The influence of hand posture on corticospinal excitability during motor imagery: A transcranial magnetic stimulation study. *Cerebral Cortex*, *14*(11), 1200–1206. 10.1093/cercor/bhh080 15142965 10.1093/cercor/bhh080

[CR53] Viviani, P., & Flash, T. (1995). Minimum-jerk, two-thirds power law, and isochrony: Converging approaches to movement planning. *Journal of Experimental Psychology: Human Perception and Performance*, *21*(1), 32–53. 10.1037/0096-1523.21.1.32 7707032 10.1037//0096-1523.21.1.32

[CR54] Wolpert, D. M., & Ghahramani, Z. (2000). Computational principles of movement neuroscience. *Nature Neuroscience*, *3*(S11), 1212–1217. 10.1038/81497 11127840 10.1038/81497

[CR55] Wolpert, D. M., Doya, K., & Kawato, M. (2003). A unifying computational framework for motor control and social interaction. *Philosophical Transactions of the Royal Society B: Biological Sciences*, *358*(1431), 593–602. 10.1098/rstb.2002.1238 10.1098/rstb.2002.1238 PMC1693134 12689384

[CR56] Zimmermann, M., Toni, I., & de Lange, F. P. (2013). Body posture modulates action perception. *The Journal of Neuroscience*, *33*(14), 5930–5938. 10.1523/JNEUROSCI.5570-12.2013 23554475 10.1523/JNEUROSCI.5570-12.2013 PMC6618930

[CR57] Zucchini, E., Borzelli, D., & Casile, A. (2023). Representational momentum of biological motion in full-body, point-light and single-dot displays. *Scientific Reports*, *13*(1), 10488. 10.1038/s41598-023-36870-2 37380666 10.1038/s41598-023-36870-2 PMC10307891

